# Supplementary material for: Machine Learning Accelerates Crystallization for Structure Determination
Source: Angew Chem Int Ed Engl. 2026 May 4;65(25):e1218503. doi: 10.1002/anie.1218503 (PMC13266958; doi:10.1002/anie.1218503)

checkCIF/PLATON report

Structure factors have been supplied for datablock(s) lcz040_auto

THIS REPORT IS FOR GUIDANCE ONLY. IF USED AS PART OF A REVIEW PROCEDURE FOR PUBLICATION, IT SHOULD NOT REPLACE THE EXPERTISE OF AN EXPERIENCED CRYSTALLOGRAPHIC REFEREE.

No syntax errors found. CIF dictionary Interpreting this report

Datablock: lcz040_auto

Bond precision:	C-C = 0.0105 A		Wavelength=1.54184
Cell:	a=12.0102(2)	b=12.2747(1)	c=23.4682(3)
	alpha=96.267(1)	beta=99.365(1)	gamma=112.281(1)
Temperature:	100 K		

	Calculated	Reported
Volume	3102.93(8)	3102.93(7)
Space group	P 1	P 1
Hall group	P 1	P 1
Moiety formula	C52 H48 Ag3 F18 N7 O12	C15 H3 Ag3 F18 N6, C37 H45 N O12
Sum formula	C52 H48 Ag3 F18 N7 O12	C52 H48 Ag3 F18 N7 O12
Mr	1628.59	1628.58
Dx,g cm-3	1.743	1.743
Z	2	2
Mu (mm-1)	8.581	8.581
F000	1616.0	1616.0
F000'	1622.96	
h,k,lmax	14,15,29	14,14,29
Nref	24826[12413]	17236
Tmin,Tmax	0.159,0.424	0.470,1.000
Tmin'	0.061	

Correction method= # Reported T Limits: Tmin=0.470 Tmax=1.000
AbsCorr = MULTI-SCAN

```
Data completeness= 1.39/0.69        Theta(max)= 73.092

R(reflections)= 0.0356( 16752)       wR2(reflections)= 0.0946( 17236)

S = 1.035                  Npar= 1690
```

The following ALERTS were generated. Each ALERT has the format
test-name_ALERT_alert-type_alert-level.
Click on the hyperlinks for more details of the test.

Alert level B

```
PLAT915_ALERT_3_B No Flack x Check Done: Low Friedel Pair Coverage         44 %
```

Author Response: Due to insufficient data. The compound crystallizes in the chiral space group, but no chiral ligand is used, and the complex is no longer chiral.

Alert level C

```
PLAT042_ALERT_1_C Calc. and Reported MoietyFormula Strings  Differ     Please Check
              Calc: C52 H48 Ag3 F18 N7 O12
              Rep.: C15 H3 Ag3 F18 N6, C37 H45 N O12
PLAT090_ALERT_3_C Poor Data / Parameter Ratio (Zmax > 18) ........       6.94 Note
PLAT213_ALERT_2_C Atom F04L            has ADP max/min Ratio .....        3.3 prolat
PLAT213_ALERT_2_C Atom F04X            has ADP max/min Ratio .....        3.2 prolat
PLAT213_ALERT_2_C Atom F052            has ADP max/min Ratio .....        3.8 prolat
PLAT220_ALERT_2_C NonSolvent   Resd 1  C   Ueq(max)/Ueq(min) Range        3.6 Ratio
PLAT220_ALERT_2_C NonSolvent   Resd 1  F   Ueq(max)/Ueq(min) Range        4.0 Ratio
PLAT222_ALERT_3_C NonSolvent Resd 1  H   Uiso(max)/Uiso(min) Range        4.8 Ratio
PLAT342_ALERT_3_C Low Bond Precision on  C-C Bonds ..............    0.01053 Ang.
PLAT352_ALERT_3_C Short  N-H (X0.87,N1.01A)  N01Q     - H01Q     .       0.75 Ang.
PLAT414_ALERT_2_C Short Intra D-H..H-X       H019     ..H02M     .       1.93 Ang.
                                                    x,y,z  =      1_555 Check
PLAT601_ALERT_2_C Unit-Cell Contains Solvent Accessible VOIDS .LE.         34 Ang**3
PLAT911_ALERT_3_C Missing FCF Refl Between Thmin & STh/L=    0.600         65 Report
               -2 13  0,  -1 13  0,   0 13  0,  -3 14  0,  -2 14  0,   2-14  1,
                3-14  1,  -1-13  1,   0-13  1,   1-13  1,   2-13  1,  -2 13  1,
               -1 13  1,   0 13  1,  -3 14  1,   2-14  2,   3-14  2,  -1-13  2,
                0-13  2,   1-13  2,   2-13  2,  -3-12  2,  -2 13  2,  -1 13  2,
                0 13  2,  -3 14  2,   2-14  3,   3-14  3,  -1-13  3,   0-13  3,
                   (   35 More Missing: see the .ckf listing file)
```

Alert level G

```
PLAT003_ALERT_2_G Number of Uiso or U(i,j)  Restrained non-H-Atoms          1 Report
PLAT004_ALERT_5_G Polymeric Structure Found with Maximum Dimension          1 Info
PLAT007_ALERT_5_G Number of Unrefined Donor-H Atoms .............           6 Report
              H00D  H019  H01J  H00E  H00I  H01A
PLAT154_ALERT_1_G The s.u.'s on the Cell Angles are Equal ..(Note)      0.001 Degree
PLAT186_ALERT_4_G The CIF-Embedded .res File Contains ISOR Records          1 Report
```

```
PLAT242_ALERT_2_G Low    'MainMol' Ueq as Compared to Neighbors of       C049 Check
PLAT242_ALERT_2_G Low    'MainMol' Ueq as Compared to Neighbors of       C04I Check
PLAT242_ALERT_2_G Low    'MainMol' Ueq as Compared to Neighbors of       C04O Check
PLAT242_ALERT_2_G Low    'MainMol' Ueq as Compared to Neighbors of       C04P Check
PLAT242_ALERT_2_G Low    'MainMol' Ueq as Compared to Neighbors of       C04T Check
PLAT242_ALERT_2_G Low    'MainMol' Ueq as Compared to Neighbors of       C053 Check
PLAT242_ALERT_2_G Low    'MainMol' Ueq as Compared to Neighbors of       C042 Check
PLAT242_ALERT_2_G Low    'MainMol' Ueq as Compared to Neighbors of       C04N Check
PLAT242_ALERT_2_G Low    'MainMol' Ueq as Compared to Neighbors of       C04Q Check
PLAT242_ALERT_2_G Low    'MainMol' Ueq as Compared to Neighbors of       C04Y Check
PLAT242_ALERT_2_G Low    'MainMol' Ueq as Compared to Neighbors of       C04Z Check
PLAT242_ALERT_2_G Low    'MainMol' Ueq as Compared to Neighbors of       C051 Check
PLAT335_ALERT_2_G Check Large C6 Ring C-C Range C01W     -C02I           0.15 Ang.
PLAT380_ALERT_4_G Incorrectly? Oriented X(sp2)-Methyl Moiety .....       C04A Check
PLAT398_ALERT_2_G Deviating  C-O-C   Angle From 120 for O007     .      108.6 Degree
PLAT398_ALERT_2_G Deviating  C-O-C   Angle From 120 for O009     .      108.6 Degree
PLAT434_ALERT_2_G Short Inter HL..HL Contact F00F     ..F00L     .       2.78 Ang.
                                                      x,y,z  =     1_555 Check
PLAT434_ALERT_2_G Short Inter HL..HL Contact F00R     ..F027     .       2.83 Ang.
                                                      x,y,z  =     1_555 Check
PLAT720_ALERT_4_G Number of Unusual/Non-Standard Labels ..........        270 Note
              Ag01    Ag02    Ag03    Ag04    Ag05    Ag06    O007    O008
              O009    O00A    F00B    F00C    O00D    H00D    O00E    H00E
              F00F    O00G    F00H    O00I    H00I    O00J    F00K    F00L
              F00M    F00N    F00O    O00P    O00Q    F00R    F00S    F00T
              F00U    F00V    O00W    F00X    O00Y    N00Z    F010    F011
              F012    O013    O014    F015    O016    F017    F018    O019
              H019    O01A    H01A    F01B    O01C    F01D    F01E    N01F
              F01G    F01H    O01I    O01J    H01J    O01K    N01L    N01M
              F01N    O01O    N01P    N01Q    N01R    N01S    N01T    N01V
              C01W    C01X    C01Y    O01Z    N020    N021    C022    H022
              C023    F024    C025    F026    F027    F028    C029    H02E
              H02F    H02G    C02A    C02B    H02B    C02C    H02H    C02D
              N02E    C02F    H02K    C02G    C02H    C02I    H02I    C02J
              H02J    C02K    C02L    C02M    H02M    C02N    C02O    H02Q
              C02P    H02P    C02Q    H02A    H02C    H02D    C02R    H02R
              C02S    C02T    H02T    C02U    H02L    H02N    H02O    C02V
              H02V    C02W    C02X    H02X    C02Y    C02Z    C030    C031
              H03N    H03O    H03P    C032    C033    H03Q    H03R    H03S
              C034    H034    C035    C036    H036    C037    H03A    H03B
              H03C    C038    H038    F039    C03A    H03T    H03V    H03W
              C03B    H03X    C03C    H03Y    C03D    C03E    C03F    H03Z
              F03G    C03H    C03I    H03     C03J    C03K    C03L    H03L
              C03M    H03D    H03E    H03F    C03N    C03O    H03G    H03H
              H03I    C03P    C03Q    C03R    C03S    C03T    C03U    H03U
              C03V    H03J    H03K    H03M    C03W    C03X    Ha      C03Y
              C03Z    C040    C041    C042    C043    C044    C045    H04A
              H04B    H04C    C046    C047    C048    H048    C049    C04A
              H04K    H04L    H04M    C04B    C04C    H04N    C04D    H04D
              C04E    C04F    C04G    H04U    C04H    C04I    C04J    H04O
              H04P    H04Q    C04K    F04L    C04M    H04E    H04F    H04G
              C04N    C04O    C04P    C04Q    C04R    C04S    H04R    H04S
              H04T    C04T    C04U    C04V    H04H    H04I    H04J    C04W
              H04W    F04X    C04Y    C04Z    C050    C051    F052    C053
              C054    H05A    H05B    H05C    H01M    H01Q
PLAT860_ALERT_3_G Number of Least-Squares Restraints .............          9 Note
PLAT910_ALERT_3_G Missing FCF Reflection(s) Below Theta(Min)[Deg]=       3.89 Note
```

```
                0  0  1,
PLAT912_ALERT_4_G Missing # of FCF Reflections Above STh/L=  0.600        590 Note
PLAT933_ALERT_2_G Number of HKL-OMIT Records in Embedded .res File          1 Note
                0  0  1,
PLAT941_ALERT_3_G Average HKL Measurement Multiplicity ..........         3.0 Low
PLAT969_ALERT_5_G The 'Henn et al.' R-Factor-gap value ..........       2.729 Note
              Predicted wR2: Based on SigI**2  3.47 or SHELX Weight  9.15
PLAT978_ALERT_2_G Number C-C Bonds with Positive Residual Density.          0 Info
```

```
   0 ALERT level A = Most likely a serious problem - resolve or explain
   1 ALERT level B = A potentially serious problem, consider carefully
  13 ALERT level C = Check. Ensure it is not caused by an omission or oversight
  31 ALERT level G = General information/check it is not something unexpected

   2 ALERT type 1 CIF construction/syntax error, inconsistent or missing data
  27 ALERT type 2 Indicator that the structure model may be wrong or deficient
   9 ALERT type 3 Indicator that the structure quality may be low
   4 ALERT type 4 Improvement, methodology, query or suggestion
   3 ALERT type 5 Informative message, check
```

Validation response form

Please find below a validation response form (VRF) that can be filled in and pasted into your CIF.

```
# start Validation Reply Form
_vrf_PLAT042_lcz040_auto
;
PROBLEM: Calc. and Reported MoietyFormula Strings  Differ     Please Check
RESPONSE: ...
;
_vrf_PLAT090_lcz040_auto
;
PROBLEM: Poor Data / Parameter Ratio (Zmax > 18) ........       6.94 Note
RESPONSE: ...
;
_vrf_PLAT213_lcz040_auto
;
PROBLEM: Atom F04L            has ADP max/min Ratio .....        3.3 prolat
RESPONSE: ...
;
_vrf_PLAT220_lcz040_auto
;
PROBLEM: NonSolvent   Resd 1  C   Ueq(max)/Ueq(min) Range        3.6 Ratio
RESPONSE: ...
;
_vrf_PLAT222_lcz040_auto
;
PROBLEM: NonSolvent Resd 1  H   Uiso(max)/Uiso(min) Range        4.8 Ratio
RESPONSE: ...
;
_vrf_PLAT342_lcz040_auto
;
PROBLEM: Low Bond Precision on  C-C Bonds ..............    0.01053 Ang.
RESPONSE: ...
;
```

```
_vrf_PLAT352_lcz040_auto
;
PROBLEM: Short  N-H (X0.87,N1.01A)  N01Q     - H01Q     .       0.75 Ang.
RESPONSE: ...
;
_vrf_PLAT414_lcz040_auto
;
PROBLEM: Short Intra D-H..H-X       H019     ..H02M     .       1.93 Ang.
RESPONSE: ...
;
_vrf_PLAT601_lcz040_auto
;
PROBLEM: Unit-Cell Contains Solvent Accessible VOIDS .LE.         34 Ang**3
RESPONSE: ...
;
_vrf_PLAT911_lcz040_auto
;
PROBLEM: Missing FCF Refl Between Thmin & STh/L=    0.600         65 Report
RESPONSE: ...
;
# end Validation Reply Form
```

It is advisable to attempt to resolve as many as possible of the alerts in all categories. Often the minor alerts point to easily fixed oversights, errors and omissions in your CIF or refinement strategy, so attention to these fine details can be worthwhile. It is up to the individual to critically assess their own results and, if necessary, seek expert advice.

PLATON version of 17/09/2025; check.def file version of 17/09/2025

---

duplicate check

No duplication found

Datablock lcz040_auto - ellipsoid plot

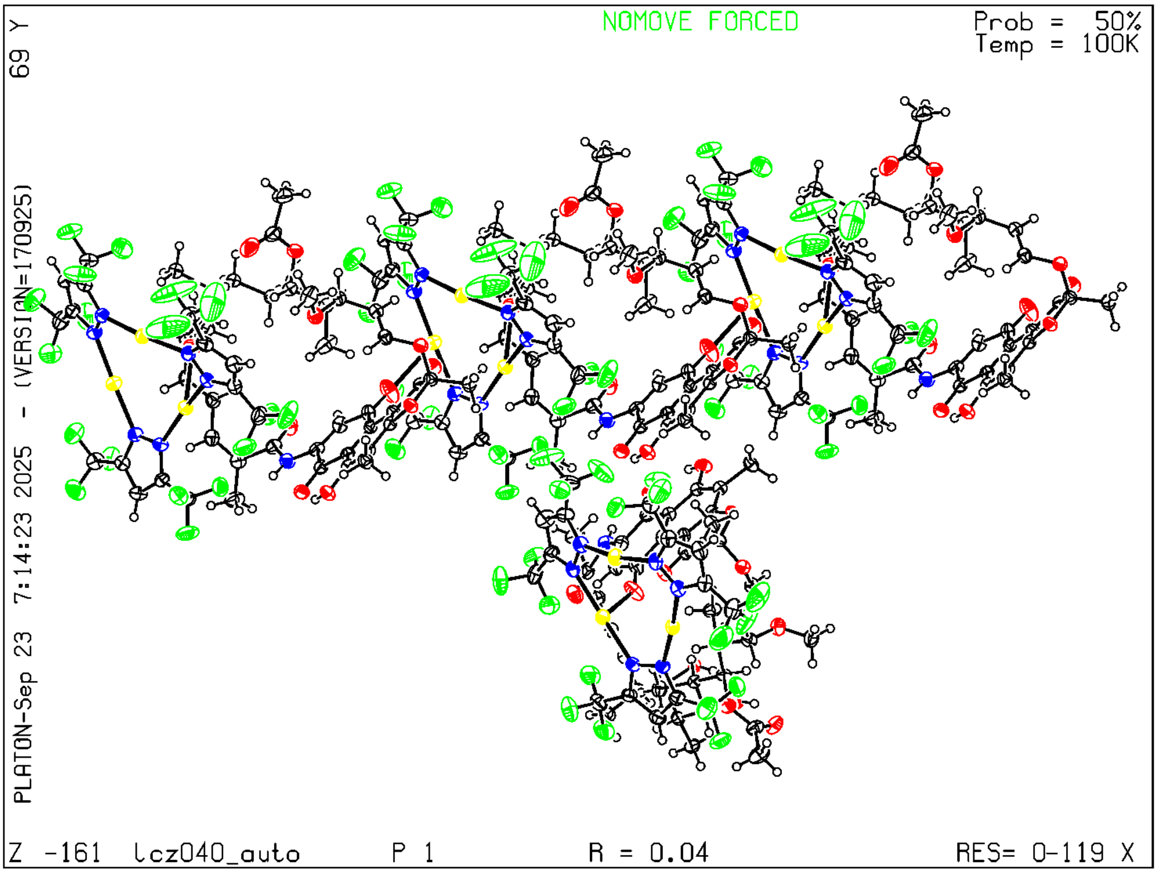

Supplement: Supplementary file 2 — Supporting File 2: anie72462‐sup‐0002‐DataFile.zip. [file ANIE-65-e1218503-s002.zip › supplementary_materials_crystallographic_data submit.zip!/cif_and_checkcif(1-114)/Ag3Pz3_109/Ag3Pz3_109_checkcif.pdf]
